# Targeting PDGF‐mediated recruitment of pericytes blocks vascular mimicry and tumor growth

**DOI:** 10.1002/path.5152

**Published:** 2018-10-30

**Authors:** Victor LJL Thijssen, Yvette WJ Paulis, Patrycja Nowak‐Sliwinska, Katrin L Deumelandt, Kayoko Hosaka, Patricia MMB Soetekouw, Anca M Cimpean, Marius Raica, Patrick Pauwels, Joost J van den Oord, Vivianne CG Tjan‐Heijnen, Mary J Hendrix, Carl‐Henrik Heldin, Yihai Cao, Arjan W Griffioen

**Affiliations:** ^1^ Angiogenesis Laboratory, Department of Medical Oncology VU University Medical Center Amsterdam The Netherlands; ^2^ Department of Radiation Oncology VU University Medical Center Amsterdam The Netherlands; ^3^ Division of Medical Oncology, GROW – School for Oncology and Developmental Biology Maastricht University Medical Center Maastricht The Netherlands; ^4^ School of Pharmaceutical Sciences University of Geneva Geneva Switzerland; ^5^ Department of Microbiology, Tumor and Cell Biology Karolinska Institute Stockholm Sweden; ^6^ Department of Microscopic Morphology, Histology, Angiogenesis Research Center Victor Babes University of Medicine and Pharmacy Timisoara Romania; ^7^ Department of Pathology Antwerp University Hospital Edegem Belgium; ^8^ Laboratory of Translational Cell and Tissue Research University of Leuven Leuven Belgium; ^9^ Department of Biology, Shepherd University Shepherdstown University WV USA; ^10^ Department of Medical Biochemistry and Microbiology, Science for Life Laboratory Uppsala University Uppsala Sweden

**Keywords:** tumor angiogenesis, imatinib, endothelial cells, melanoma, Ewing sarcoma, perivascular cells, vasculogenic mimicry, vessel stabilization, cancer

## Abstract

Aggressive tumor cells can adopt an endothelial cell‐like phenotype and contribute to the formation of a tumor vasculature, independent of tumor angiogenesis. This adoptive mechanism is referred to as vascular mimicry and it is associated with poor survival in cancer patients. To what extent tumor cells capable of vascular mimicry phenocopy the angiogenic cascade is still poorly explored. Here, we identify pericytes as important players in vascular mimicry. We found that pericytes are recruited by vascular mimicry‐positive tumor cells in order to facilitate sprouting and to provide structural support of the vascular‐like networks. The pericyte recruitment is mediated through platelet‐derived growth factor (PDGF)‐B. Consequently, preventing PDGF‐B signaling by blocking the PDGF receptors with either the small tyrosine kinase inhibitor imatinib or blocking antibodies inhibits vascular mimicry and tumor growth. Collectively, the current study identifies an important role for pericytes in the formation of vascular‐like structures by tumor cells. Moreover, the mechanism that controls the pericyte recruitment provides therapeutic opportunities for patients with aggressive vascular mimicry‐positive cancer types. © 2018 The Authors. *The Journal of Pathology* published by John Wiley & Sons Ltd on behalf of Pathological Society of Great Britain and Ireland.

## Introduction

Aggressive tumor cells are known to form vascular‐like networks that are not lined by endothelial cells, a process referred to as vascular mimicry (VM) 1, 2. These networks contribute to tumor blood flow and tumor progression 3, 4, 5, 6. Recent findings suggest that VM can also be a driving force in tumor metastasis 7 and induction of resistance to angiostatic compounds 8. The presence of VM in human tumor tissues correlates with poor clinical outcome 9, which has initiated efforts to therapeutically target VM. Unfortunately, the vascular‐like structures that are formed by VM‐positive tumor cells are hardly sensitive to classical angiogenesis inhibitors 6, 10, 11 and angiostatic treatment has even been shown to induce VM development 12, 13. Thus, a better understanding of the mechanisms that drive VM is pivotal as it can provide new therapeutic opportunities.

Previous research has linked VM to epithelial‐to‐mesenchymal transition (EMT) 14, 15 as well as to enrichment of a cancer stem cell (CSC)‐like phenotype 3, 6. The enhanced tumor cell plasticity that is associated with both of these processes allows VM‐positive (VM^+^) tumor cells to acquire endothelial cell‐like traits and to mimic blood vessel formation 1, 16. Therefore, several molecular regulators involved in EMT and CSCs are considered as potential druggable targets for VM, e.g. Twist 1 17, CD44/c‐MET 18, and Nodal/Notch4 19. A drug affecting the latter pathway is now in clinical testing 20. These drugs target the EMT/CSC phenotype, but it is still poorly understood as to what extent VM^+^ tumor cells phenocopy different steps of physiological angiogenesis and whether this provides additional opportunities for therapeutic strategies.

An important step in angiogenesis is the recruitment of pericytes to the newly formed capillary sprouts. Pericytes have been found to exert multiple roles in the vasculature. For example, the inability to recruit pericytes was found to result in aberrant vascular remodeling and vessel regression 21. At the same time, pericytes have also been linked to vascular remodeling by promoting vessel pruning 22. More recent papers suggest that pericyte–endothelial cell interactions are also directly involved in controlling VEGF signaling which affects endothelial sprouting 23 and the formation of a functional blood–retina barrier 24. An important function of pericyte–endothelial cell interactions in angiogenesis is promoting vascular maturation by inducing the production and deposition of matrix components such as fibronectin, collagen, and laminin 25, 26. Since the vascular networks formed by VM tumor cells are rich in extracellular matrix depositions, we hypothesized that VM^+^ tumor cells phenocopy this mechanism of vessel maturation by recruitment of pericytes. In this report, we provide evidence that VM^+^ tumor cells indeed attract pericytes, which supports the formation and stabilization of vascular networks. The pericyte recruitment is mediated by tumor cell‐derived PDGF‐B, and targeting the PDGF signaling axis prevents the formation of VM networks and suppresses tumor growth. Collectively, the current data show that VM‐positive tumors exploit pericytes for structural support of vascular‐like networks, which provides a therapeutic opportunity for patients with aggressive cancers.

## Materials and methods

### Cell culture

The human cutaneous (c81‐61, C8161) and uveal (OCM‐1) melanoma cell lines have been described previously 2, 27 and were maintained in RPMI‐1640 (Lonza, Breda, The Netherlands) supplemented with 10% fetal bovine serum gold (FBS; Fisher Scientific, Landsmeer, The Netherlands). The Ewing sarcoma cell lines EW7 and RDES 5 were maintained in RPMI‐1640 (Lonza) supplemented with 10% fetal bovine serum gold (FBS; Fisher Scientific) and 2 mm l‐glutamine. Human perivascular cells from the vena saphena magna (HVSCs) were isolated in accordance to Dutch guidelines for the secondary use of material (https://www.federa.org) and maintained in advanced Dulbecco's modified Eagle's medium (Gibco; Fisher Scientific) supplemented with 10% FBS, 2 mm glutamine, and 1% penicillin/streptomycin (Lonza). Human umbilical vein endothelial cells (HUVECs) and RF24 cells (EC‐RF24; immortalized HUVECs) were cultured as described previously 28. All cells were cultured at 37 °C under a humidified atmosphere containing 5% CO_2_. Cell cultures were routinely confirmed to be negative for mycoplasma infection.

### PDGFBB ELISA

Quantification of PDGFBB protein levels accumulating in the medium of confluent cell cultures was carried out using the Quantikine human PDGFBB immunoassay (R&D Systems, Abingdon, UK). In brief, cells were cultured for 5 days in medium containing 5% FBS. Conditioned medium was analyzed for human PDGFBB expression according to the manufacturer's protocol. Optical density was measured at 450 nm with wavelength correction set to 540 nm using a Tecan SpectraFluor Absorbance Microplate Reader (Tecan Group, Männedorf, Switzerland).

### Matrigel assay

Wells of a 96‐well plate were coated with 40 μl of growth factor reduced (GFR−) Matrigel (BD Biosciences, Vianen, The Netherlands) for 30–60 min at 37 °C. VM^+^ melanoma cells were seeded onto the Matrigel at a density of 20 000 cells per well in a volume of 50 μl. Two thousand additional cells, being either VM^+^ or VM^−^ tumor cells, immortalized endothelial cells (RF‐24), or perivascular cells were added to the wells in a volume of 25 μl. Network formation was analyzed after 24 and 96 h. Images were captured using a Leica DMI3000B microscope 29.

### Matrigel invasion assay

Perivascular cell invasion was measured in a 24‐well plate Transwell system (BD Biosciences) containing fluorescence blocking 8.0 μm pore polycarbonate filter inserts (HTS FluoroBlok Insert, BD Biosciences). Inserts were coated overnight with 100 μl of Matrigel (50 μg/ml in PBS) (Sigma‐Aldrich, Zwijndrecht, The Netherlands). Per insert, 50 000 cells were seeded in serum‐free advanced DMEM containing l‐glutamine. Serum‐free medium enriched with concentrated melanoma cells conditioned medium (10‐fold diluted) or PDGFBB (100 ng/ml) was added to the bottom compartment. For tyrosine kinase inhibition, 5 μm imatinib or 5 μm erlotinib was added to both the insert and the bottom compartment. Cells were allowed to invade for 8 h, after which invading cells were fixed with 4% paraformaldehyde and permeabilized with 0.1% Triton‐X. Subsequently, cells were stained with rhodamine phalloidin (Invitrogen, Fisher Scientific) for visualization of F‐actin. Cells were imaged by fluorescence microscopy using a Leica DFC 345 FX microscope 29.

### *In vivo* murine models

All animal experiments were approved by the local animal ethics committee. In brief, 1 × 10^6^ human C8161 or OCM‐1 melanoma cells were injected subcutaneously into the flanks of Swiss/nude mice 5. Treatment with imatinib (STI‐571, 50 mg/kg, i.p.) was performed daily; treatment with antibodies against PDGFα or PDGFβ was performed by i.p. injections of 100 μg, once weekly. Tumor growth was monitored by daily measurement. At the end of the experiments, tumors were excised and processed for histological analyses. The generation of B16F10 cells with stable expressing of PDGF and the animal experiments using these murine melanoma cells have been described previously 30.

### Statistical analysis

All data are expressed as mean values ± standard error of the mean (SEM) unless indicated otherwise. Statistical analyses were performed using Student's *t*‐test (parametric) or the Mann–Whitney *U*‐test (nonparametric) in the case of two conditions. When multiple conditions were compared, a one‐way ANOVA with Bonferroni's multiple comparison test was used. The tumor growth curves and network stabilization data were analyzed using a two‐way ANOVA with Bonferroni's *post hoc* test. All statistical analyses were performed using SPSS 20.0.0 (IBM, Amsterdam, The Netherlands) or in GraphPad Prism 7.0 (Graphpad Software Inc, La Jolla, CA, USA). *P* values less than or equal to 0.05 were considered statistically significant.

## Results

### Pericytes line up with VM structures

**Figure 1:**
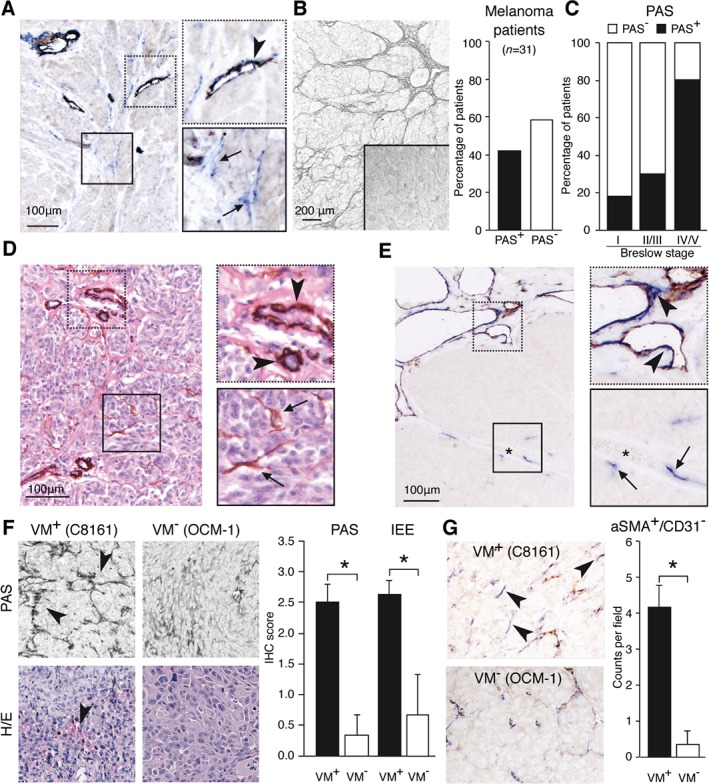
Vascular‐like structures in aggressive VM^+^ tumors contain perivascular cells that are not associated with regular blood vessels. (A) Double staining for the endothelial cell marker CD31 (brown) and the perivascular marker αSMA (blue) in human melanoma tissue. The upper right panel shows αSMA^+^ cells around a CD31^+^ blood vessel (arrowhead). The lower right panel shows the presence of αSMA^+^ cells not associated with CD31^+^ endothelial cells (arrows). (B) Representative picture of PAS staining of VM‐characteristic patterned vascular networks in human melanoma tissue. The bar graph shows the percentage of melanoma patients showing this PAS^+^ staining. (C) Percentage PAS^+^ and PAS^−^ tumor tissues in melanoma patients classified according invasive activity, based on Breslow staging. (D) αSMA staining of a melanoma tissue section counterstained with hematoxylin and eosin. The upper right panel shows αSMA^+^ cells around blood vessels (arrowheads). The lower right panel shows the presence of αSMA^+^ cells associated with vasculogenic matrix networks (arrows). (E) Similar staining to that in A on Ewing sarcoma tissue. Asterisk indicates a VM‐characteristic blood lake. (F) PAS staining (upper panels) and hematoxylin and eosin staining (H/E; lower panels) of C8161 tumors (left panels) and OCM‐1 tumors (right panels). The bar graph shows quantification (± SEM) of the PAS loops and intratumoral extravascular erythrocytes (IEE) in OCM‐1 (*n* = 4) and C8161 (*n* = 5) tumors. *p < 0.05, Student's *t*‐test. (G) Double staining similar to that in A of OCM‐1 (*n* = 4) and C8161 (*n* = 5) tumors. Arrowheads indicate αSMA^+^ cells not associated with CD31^+^ endothelial cells. The bar graph shows the number of αSMA^+^ cells (± SEM) per microscopic field. *p < 0.05, Student's *t*‐test.

To explore whether pericytes contribute to VM, we stained a series of primary human cutaneous melanoma tissues, a tumor type that is known to frequently display VM, using different pericyte markers, i.e. α‐smooth muscle actin (αSMA), neural/glial antigen 2 (NG2), and desmin. This revealed that pericytes were not exclusively associated with blood vessels but also appeared distant from endothelial cells **(**Figure 1A and supplementary material, Figure S1 **)**. To determine whether these cells line up with VM structures, both αSMA and periodic acid–Schiff (PAS) staining was performed. PAS‐positive (PAS^+^) loops, which are indicative of VM, were observed in 42% of the tumors **(**Figure 1B**)**. In line with previous studies 2, 31, 32, a higher incidence of PAS^+^ loops was associated with increased tumor aggressiveness **(**Figure 1C**)**. The same was observed for another characteristic of VM, i.e. the presence of intratumoral extravascular erythrocytes (IEEs) 31 (supplementary material, Figure S2 **)**. Importantly, PAS^+^ tissues frequently stained positive for αSMA within the extracellular matrix networks that lined the tumor cells **(**Figure 1D**)**. In contrast, αSMA^+^ cells that were not associated with blood vessels were never observed in PAS^−^ tumors or regions. The commonality of these observations was confirmed in a series of human Ewing sarcoma tissues, in which VM is characterized by tumor cell‐lined blood lakes 5. In these tissues, αSMA^+^ cells were again observed in VM^+^ regions devoid of CD31^+^ endothelial cells **(**Figure 1E**)**. To further confirm these findings, VM^−^ and VM^+^ melanoma tumors were grown subcutaneously in mice, as described previously 5. Similarly as in patients, the VM^+^ melanoma tumors displayed a significantly increased incidence of both PAS loops and IEEs, compared with poorly aggressive VM^−^ tumors **(**Figure 1F**)**. Double staining again showed the presence of αSMA^+^ pericytes that were not associated with CD31^+^ endothelial cells in VM^+^ tumors. This was rarely observed in VM^−^ tumors **(**Figure 1G**)**. Of note, there was no difference in normal blood vessels between the VM^+^ and VM^−^ tumors (supplementary material, Figure S3). Collectively, these observations in experimental and clinical melanoma tumors suggest that vascular‐forming tumor cells in aggressive VM^+^ cancers attract pericytes.

### Pericytes facilitate vascular‐like structure formation by tumor cells

**Figure 2:**
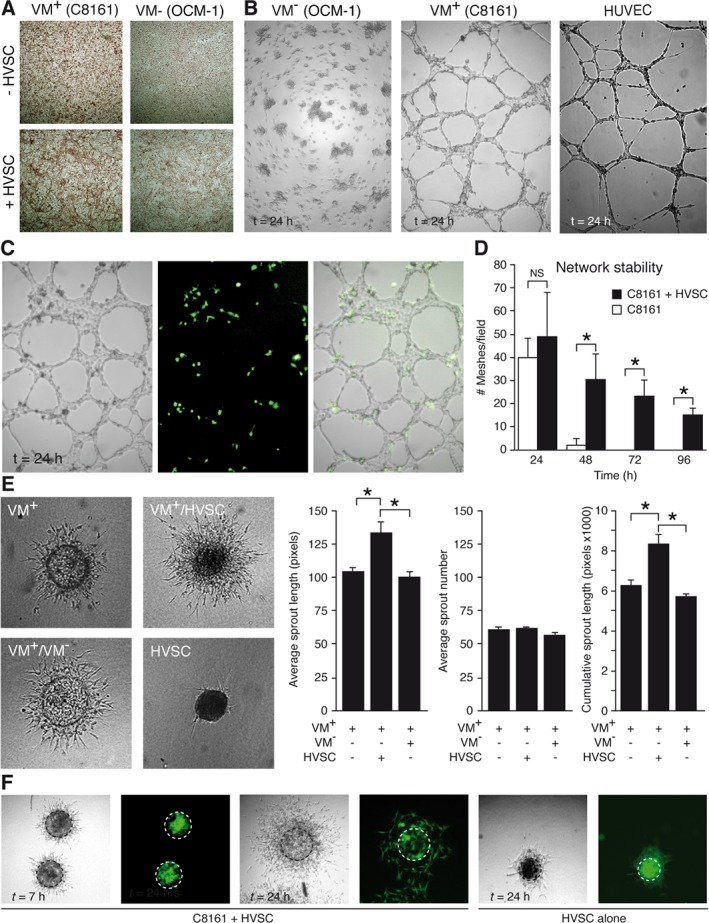
Perivascular cells facilitate the formation and stabilization of vascular‐like structures formed by VM^+^ tumor cells. (A) PAS staining of confluent cultures of VM^+^ C8161 melanoma cells (left panels) and VM^−^ OCM‐1 melanoma cells in the absence (upper panels) or presence (lower panels) of human vena saphena magna cells (HVSC). (B) Network formation on Matrigel by VM^−^ and VM^+^ melanoma cells as well as endothelial cells (HUVEC). (C) Network formation on Matrigel by VM^+^ melanoma cells in the presence of CFSE‐labeled HVSCs. The left panel shows a bright‐field image of the networks. The middle panel shows fluorescence microscopy. Only the HSVCs were CFSE‐labeled perivascular cells (green). The right panel shows an overlay of both pictures, showing co‐localization of the perivascular cells with the networks. (D) Quantification of the number of meshes formed by VM^+^ melanoma cells in the presence or absence of perivascular cells in time. *p < 0.05, two‐way ANOVA. NS = not significant. (E) Analysis of sprouting by VM^+^ melanoma cell spheroids in a 3D collagen matrix in the absence (upper left) or presence of HVSCs (upper right). Sprouting without HVSCs but with VM^−^ cells as an additional source is shown in the lower left image. HVSCs do not sprout by themselves (lower right image). Bar graphs show quantification of the sprouting (± SEM). *p < 0.05, one‐way ANOVA. (F) Representative pictures showing localization of CFSE‐labeled perivascular cells (green) in specific spheroids in time.

Previously, it has been shown that co‐culture of endothelial cells with pericytes induces the expression of basement membrane proteins 25. To explore the role of pericytes in vascular‐like network formation by tumor cells, human VM^+^ and VM^−^ cells were cultured in the presence or absence of pericytes. Co‐culture of different VM^+^ tumor cells with pericytes (human vena saphenous cells; HVSCs) resulted in PAS^+^ matrix depositions that were reminiscent of the PAS^+^ loops observed in human cancer tissues **(**Figure 2A and supplementary material, Figure S4A**)**. This was not observed when (1) either cell type was cultured alone, (2) VM^+^ cells were co‐cultured with non‐pericytes, or (3) VM^−^ cells were co‐cultured with pericytes (supplementary material, Figure S4B). The functional contribution of the pericytes to VM was further explored by co‐culture of HVSCs with different VM^+^ or VM^−^ tumor cells on Matrigel. As described previously 2, 5, we observed that VM^−^ tumor cells show no intercellular organization when grown on extracellular matrix, whereas VM^+^ tumor cells have the ability to form honeycomb‐like networks, similar to endothelial cells **(**Figure 2B and supplementary material, Figure S5A). Addition of CFSE‐labeled HVSCs suggested their participation in the VM^+^ networks as the cells exclusively localized with the networks formed by the VM^+^ cells up to 96 h **(**Figure 2C and supplementary material, Figure S5B). While their presence did not affect the network complexity, i.e. the number of meshes, the stability of the VM networks was significantly prolonged by HVSCs (Figure 2D). This was a pericyte‐dependent effect on VM^+^ cells, as neither the addition of non‐pericytes to VM^+^ tumor cells nor the combination of pericytes with VM^−^ cells resulted in the induction or stabilization of the networks (supplementary material, Figure S5C). Moreover, the effects partly relied on cell–cell contact as no networks were formed when normal medium was replaced with conditioned medium from HVSCs (supplementary material, Figure S5D). Besides network stabilization, HVSCs also facilitated the sprouting of VM^+^ tumor cells from spheroids embedded in a 3D collagen matrix **(**Figure 2E**)**. Similar to the case of the 2D network formation, this effect was dependent on HVSCs, as the combination of VM^+^ melanoma cells with other cells did not have any contributing effect **(**Figure 2E**)**. Of note, the pericytes did not increase the sprout number but resulted in increased sprout length. Labeling the HVSCs showed that the cells remained at the center of the spheroid in the initial phase of sprouting, after which they migrated along the sprouting tumor cells **(**Figure 2F**)**. Collectively, these data suggest that VM^+^ cells have acquired the ability to recruit pericytes in order to facilitate sprouting and to stabilize the vascular‐like networks.

### Pericyte recruitment in VM is mediated through PDGF‐B signaling

**Figure 3:**
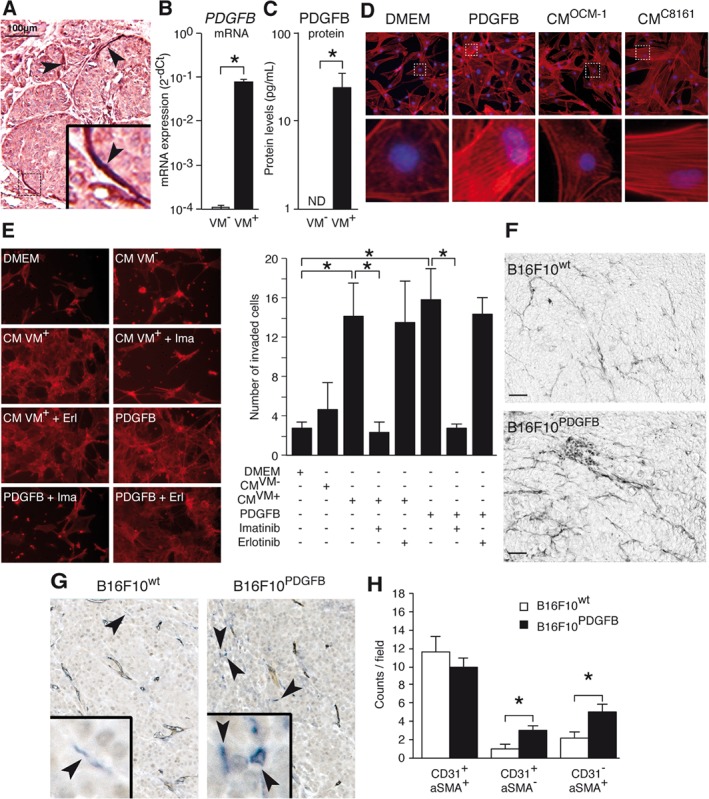
PDGF‐B induces increased pericyte recruitment by vasculogenic tumor cells *in vitro* and *in vivo*. (A) Staining for PDGF‐B (brown) in a human melanoma tissue section counterstained with hematoxylin and eosin. Arrowheads indicate regions with high PDGF‐B expression lining VM‐characteristic PAS loops. (B) RT‐qPCR data for *PDGFB* mRNA expression levels (± SEM) in cutaneous melanoma VM^−^ and VM^+^ cells (c81‐61, C8161). *p < 0.05, Student's *t*‐test. (C) PDGF‐B protein levels (± SEM) determined by ELISA in culture medium of VM^−^ and VM^+^ cells. *p < 0.05, Student's *t*‐test. (D) Representative images of HVSCs cultured in plain medium (DMEM), plain medium containing recombinant PDGF‐B (100 ng/ml), or conditioned medium (CM) of VM^−^ cells (OCM‐1) and VM^+^ cells (C8161). Cells were stained with rhodamine phalloidin (red) to visualize stress fibers. Lower panels show single‐cell magnifications. Nuclei were visualized with DAPI staining (blue). (E) Images of HSVC invasion into Matrigel towards different stimuli. DMEM = plain medium; CM VM^−^/VM^+^ = conditioned medium of either VM^−^ or VM^+^ cells; Ima = imatinib; Erl = erlotinib. Staining similar to that in D. Bar charts showing quantification of the number of invading cells (± SEM) under the different conditions. *p < 0.05, one‐way ANOVA. (F) Representative pictures of PAS staining on control transfected (mock; upper panel) and PDGF‐B transfected (PDGFB; lower panel) B16F10 tumors 17 days after tumor injection in the flanks of C57B16/J mice. (G) Double staining for the endothelial cell marker CD31 (brown) and the pericyte marker αSMA (blue) in tumors of mock transfected (left panel) and PDGF‐B transfected (right panel) B16F10 cells. Arrowheads indicate αSMA^+^ perivascular cells that are not associated with CD31^+^ blood vessels. (H) Quantification (± SEM) of perivascular cells associated with endothelial cells in regular blood vessels (CD31^+^/αSMA^+^), blood vessels without perivascular cells (CD31^+^/αSMA^−^), and perivascular cells not associated with endothelial cells (CD31^−^/αSMA^+^). *p < 0.05, one‐way ANOVA.

A key factor involved in the activation and recruitment of pericytes during normal angiogenesis is platelet‐derived growth factor B (PDGF‐B). Normally, PDGF‐B is secreted by sprouting endothelial cells, while pericytes express the PDGF receptors 33, 34. Interestingly, elevated expression of PDGF‐B or PDGF receptors by tumor cells has already been related to increased tumor malignancy 35, 36. Thus, we hypothesized that tumor cell‐derived PDGF‐B was responsible for pericyte recruitment in VM^+^ tumor areas. In support of this, high PDGF‐B expression in human cutaneous melanoma tissues was mainly observed in tumor cells lining the VM‐characteristic PAS^+^ loops (Figure 3A). Furthermore, *PDGF‐B* mRNA expression was found to be elevated several orders of magnitude in VM^+^ cell lines, compared with VM^−^ cells **(**Figure 3B and supplementary material, Figure S6A). This was verified by ELISA, demonstrating that the culture medium of VM^+^ melanoma cells contained PDGF‐B protein at concentrations ranging from 20 to 35 pg/ml, while in the culture medium of VM^−^ cells no PDGF‐B protein was detectable (Figure 3C and supplementary material, Figure S6B). The elevated expression appeared to be an intrinsic feature of VM^+^ cells as neither hypoxia, network formation, nor the interaction with pericytes induced an additional increase in the PDGF‐B levels (supplementary material, Figure S6C–F). The functional relevance of PDGF‐B expression was revealed by culturing HVSC pericytes in the presence of conditioned medium of VM^+^ cells or recombinant PDGF‐B. Both culture conditions induced the cellular organization of actin stress fibers in HVSCs, indicative of cell activation and mobility **(**Figure 3D and supplementary material, Figure S7). Indeed, the migration of pericytes was stimulated by both conditioned medium of VM^+^ cells and recombinant PDGF‐B, but not by conditioned medium of VM^−^ cells **(**Figure 3E**)**. The enhanced migration was dependent on PDGF‐B signaling, as it could be inhibited by the small molecule PDGFR inhibitor imatinib but not by the EGFR inhibitor erlotinib **(**Figure 3E**)**. To substantiate these findings, we next assessed whether PDGF‐B expression by VM^+^ tumor cells increases vascular network stabilization *in vivo*. For this, we used B16F10 murine melanoma cells, as these are able to form VM‐characteristic patterned networks on Matrigel *in vitro* (supplementary material, Figure S8A) and we have described previously that genetically modified B16F10 cells with elevated PDGF‐B expression (B16F10^PDGFB^) show enhanced tumor growth *in vivo* 30. PAS staining of these tumors indicated more matrix networks lined by tumor cells in B16F10^PDGFB^ tumors than in B16F10 wild‐type tumors (B16F10^wt^), indicative of increased VM in B16F10^PDGFB^ tumors **(**Figure 3F**)**. Moreover, additional immunohistochemical staining indicated that B16F10^PDGFB^ tumors displayed significantly more αSMA^+^ cells that were not associated with CD31^+^ blood vessels **(**Figure 3G,H**)**. Of note, the total microvessel density in B16F10^wt^ and B16F10^PDGFB^ was not significantly different, as previously described 30 (supplementary material, Figure S8B). All of these findings suggest that the increased pericyte recruitment by VM^+^ tumor cells *in vitro* and *in vivo* is mediated by PDGF‐B.

### Targeting PDGF signaling has therapeutic value in VM

**Figure 4:**
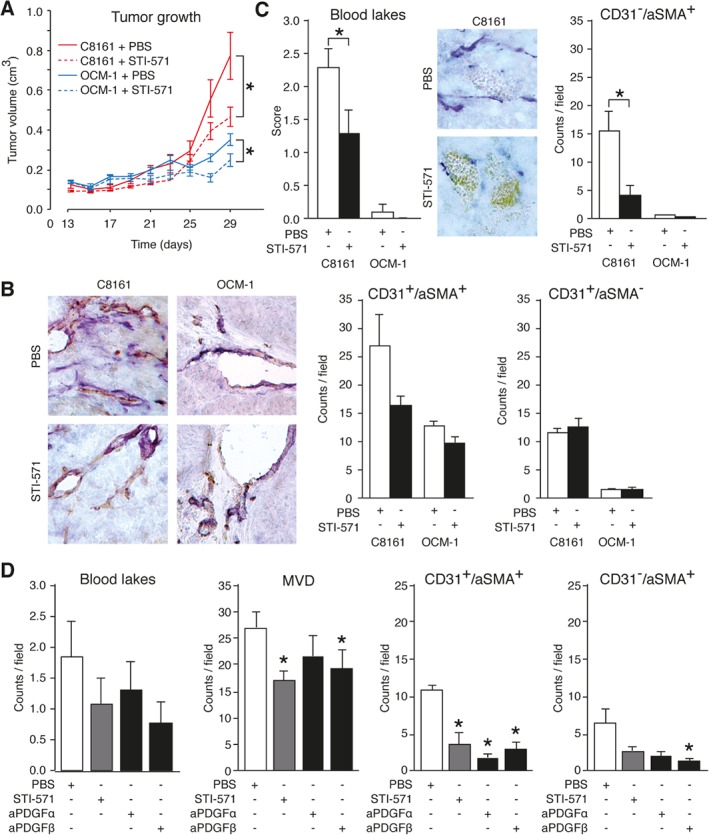
Blocking of the PDGF receptor signaling axis has therapeutic value. (A) Growth curves of VM^+^ (C8161) and VM^−^ (OCM‐1) xenograft tumors treated with or without the PDGF receptor inhibitor imatinib (STI‐571). *p < 0.05, two‐way ANOVA. (B) Double staining for the endothelial cell marker CD31 (brown) and the pericyte marker αSMA (blue) in tumor tissues from VM^+^ (C8161) and VM^−^ (OCM‐1) xenograft tumors treated with or without the PDGF receptor inhibitor (STI‐571). The bar charts show quantification of blood vessels (CD31^+^) associated with (αSMA^+^) or without (αSMA^−^) perivascular cells. (C) Similar to B for characteristics of VM, i.e. blood lakes and perivascular cells not associated with blood vessels (CD31^−^/αSMA^+^). *p < 0.05, Student's *t*‐test. (D) Quantification of blood lakes, microvessel density (MVD), blood vessels associated with perivascular cells (CD31^+^/αSMA^+^), and perivascular cells not associated with blood vessels (CD31^−^/αSMA^+^), respectively, in VM^+^ xenograft tumors treated with STI‐571, anti‐PDGFRα antibody or anti‐PDGFRβ antibody. *p < 0.05, one‐way ANOVA.

Since VM^+^ tumors are relatively insensitive to angiostatic drugs 10 and angiostatic therapy might even induce VM 12, 13, 17, 37, we explored whether targeting PDGF‐B could prevent vascular formation by VM^+^ tumor cells. To that end, human VM^+^ and VM^−^ melanoma tumor cells were grafted in SCID mice. Over 29 days, the VM^+^ tumor volumes were found to progress more rapidly compared with VM^−^ tumors (supplementary material, Figure S9A**)**. In agreement with their more aggressive phenotype, the VM^+^ tumors were characterized by an increased microvessel density and by the presence of VM‐associated blood lakes (supplementary material, Figure S9B). As expected, treatment with STI‐571 (imatinib mesylate) to block PDGF receptor signaling hampered the growth of both VM^+^ and VM^−^ tumors **(**Figure 4A**)**. In the VM^+^ tumors, the reduced growth was accompanied by a decreased microvessel density which was associated with a reduction in αSMA^+^ blood vessels **(**Figure 4B and supplementary material, Figure S9C**)**. In addition, STI‐571 treatment significantly reduced the amount of blood lakes as well as the number of αSMA^+^ cells (pericytes) not associated with blood vessels **(**Figure 4C**)**. Comparable results were observed in an additional mouse model, where xenograft VM^+^ tumors were treated with blocking antibodies targeting either PDGF receptor (PDGFR) α or PDGFRβ **(**Figure 4D and supplementary material, Figure S9D**)**. Taken together, these data suggest that targeting the VM phenotype of tumor cells by interfering with the recruitment of pericytes through blocking of the PDGF receptor signaling has therapeutic value.

## Discussion

The ability of malignant cells to form functional vascular‐like networks is associated with increased tumor aggressiveness and poor patient outcome 9. The presence of tumor cell‐lined vessels in cancer tissue is referred to as vascular mimicry (VM) 2 and the formation of such structures has been linked to the acquisition of endothelial cell‐like behavior by plastic tumor cells 16. However, to what extent VM^+^ tumor cells are able to phenocopy the different steps of physiological angiogenesis is largely unknown. The present study provides evidence that VM^+^ tumor cells not only mimic vessel sprouting and tube formation but also mimic the process of blood vessel maturation and normalization by recruiting pericytes. This is an important step in vessel formation as it has been shown in physiological angiogenesis that the inability to recruit pericytes to the endothelium results in aberrant vascular remodeling and vessel regression 21, 23, 24. Also in tumor angiogenesis, pericytes have been shown to provide a certain degree of vessel integrity and function 38, 39. In line with this, our current observations show that pericytes stabilize the vascular networks formed by VM^+^ cells. Our data also suggest that pericytes are not involved in the initiation of vessel sprouting but that their recruitment follows the process of sprout formation. This confirms previous findings 21 but is in contrast with a recent report showing that pericytes promote endothelial sprouting by controlling VEGF signaling 23. Most likely, the sprout formation by VM^+^ tumor cells relies less on VEGF signaling, compared with endothelial cell sprouting. Nevertheless, our data do suggest that VM^+^ cells have acquired the ability to promote vessel maturation using mechanisms comparable to those of normal endothelial cells. Further support for this phenomenon is the observation that culturing VM^+^ tumor cells in the presence of pericytes results in increased deposition of PAS‐reactive matrix proteins. Matrix‐rich deposits are a characteristic feature of VM. Apparently, pericytes stimulate matrix production by VM^+^ tumor cells. This is in line with the observation that pericyte–endothelial cell interactions stimulate the endothelial production and deposition of matrix components such as fibronectin, collagen, and laminin 25. Collectively, our data show that VM^+^ tumor cells are able to mimic different steps of vessel maturation by recruiting and interacting with pericytes.

Our *in vivo* studies revealed increased numbers of pericytes in VM^+^ tumors compared with VM^−^ tumors. These increased numbers could result from increased proliferation of pericytes in response to elevated levels of PDGF‐B 33. In addition, it has been shown that specific pericyte precursor cells can be recruited into tumors, where they further differentiate into pericytes and facilitate tumor angiogenesis 39, 40. These precursor cells are PDGFRβ^+^, and their recruitment and differentiation involve paracrine signaling by endothelial cells that is mediated through the PDGF‐B/PDGFRβ axis 39. More recently, it was shown that PDGF‐B/PDGFRβ signaling is also involved in the recruitment of mesenchymal stem cells and their subsequent differentiation into pericytes that promote tumor vasculogenesis 41. In addition, Shenoy *et al* have shown that transdifferentiated tumor cells can present with pericyte features, which also involves PDGF signaling 42. While we did not explore the origin of pericytes in the present study, we extend these previous findings by showing that pericyte recruitment is exploited by aggressive VM^+^ tumors during vascular network formation that is independent of regular angiogenesis or vasculogenesis. In addition, we confirm the importance of the PDGF‐B/PDGFRβ signaling axis in this process. These findings highlight a key role for pericytes in distinct mechanisms of vessel formation.

An important finding of the present study is that targeting the PDGF‐B signaling axis showed a therapeutic benefit in VM^+^ tumor models. Previously, targeting the PDGF‐B/PDGFRβ signaling axis has been shown to hamper tumor growth by inhibition of tumor vascularization 43, 44, 45. While this was confirmed in our VM^−^ tumor models, anti‐PDGFRβ treatment also reduced the VM‐characteristic vascular structures in different VM^+^ tumor models. This observation provides opportunities for the treatment of patients with aggressive VM^+^ tumors that respond poorly to anti‐angiogenic drugs 10. This is especially the case as targeting classical pro‐angiogenic signaling pathways, like VEGF signaling, has been shown to promote VM which might contribute to resistance to angiostatic therapy 12, 13, 17, 37, 46, 47. Moreover, the induction of VM might also underlie the observation that angiostatic therapy promotes tumor invasion and metastasis 37. Thus, alternative strategies are required to block vascular network formation by VM^+^ cells. In recent years, increased insights into the molecular mechanisms underlying VM have already provided druggable targets, e.g. molecular regulators of EMT/CSCs such as Notch4 19, Twist4 17, YAP1 48, and CD44/c‐Met 18. For some of these targets, investigational drugs are available 49. For example, a novel compound that has Nodal and Notch4 as major targets and that blocks VM *in vitro* is under clinical development 20.

It should be noted that the development of treatment strategies to prevent VM provides a challenge for both researchers and clinicians. Vascular mimicry is mainly associated with late‐stage tumors and metastases, while it is less frequently observed in early‐stage cancers. Thus, the effects of VM inhibitors on tumor growth will probably be confined to a limited group of patients, especially when applied as first‐line treatment or monotherapy. Instead, the most effective scenario would comprise VM inhibitors in combination with conventional or targeted (angiostatic) treatment regimens. In addition, it will be important to assess the degree of VM prior to treatment to exclude drug failure due to a lack of direct anti‐tumor activity in small groups of patients.

The current results suggest that the PDGF‐B/PDGFRβ signaling axis also constitutes a promising target to interfere with VM, as was recently also suggested for PDGF‐C 50. Interestingly, we show that this inhibition can be achieved with imatinib, a clinically available drug. Imatinib mesylate is a tyrosine kinase inhibitor that is FDA‐approved for the treatment of chronic myelogenous leukemia (CML) and gastrointestinal stromal tumors (GISTs). Apart from targeting the BCR–Abl oncoprotein and c‐KIT (CD117), which are driving kinases in CML and GISTs, respectively, imatinib also blocks PDGFRβ 51. The latter activity has previously been exploited to interfere with pericyte recruitment and function during tumor growth 41, 43. Moreover, off‐target effects of imatinib appear to benefit an adequate anti‐tumor immune response 52. Together with the inhibitory activity at the level of VM, it could be argued that the use of imatinib should not be restricted to CML and GISTs. On the other hand, it has also been demonstrated that targeting the PDGF signaling axis can lead to hypoxia‐induced EMT and subsequent metastasis 53, 54. This illustrates the dilemma of angiostatic therapy, as comparable observations have been reported using different angiostatic drugs 12, 13, 17, 37. Although such observations argue against targeting PDGF, our current data show a clear therapeutic benefit which is in line with many other reports 44, 55. This is most likely explained by the fact that in tumors that have already undergone EMT, such as VM^+^ tumors 14, 15, targeting the PDGF signaling axis will be a way to decrease tumor cell aggressiveness. In line with this, our results did not identify an effect of hypoxia on PDGFB expression in VM^+^ cells.

A limitation of the present study is that the VM^+^ and VM^−^ cells in some comparisons were not always from the same source, e.g. cutaneous or uveal. Thus, a different phenotype might be related to genetic differences between the cell lines. However, we consistently saw comparable responses in both human and murine melanoma cell lines, as well as in human Ewing sarcoma cell lines. In addition, similar observations were made in human tumor samples from both melanoma and Ewing sarcoma cancer patients. Thus, we are confident that the observations are related to the specific VM phenotype. Nevertheless, future studies in VM^+^ and VM^−^ cells that are derived from the same parental cell source will be valuable to confirm our current observations.

Furthermore, we used αSMA‐positive staining as a marker for pericytes, although myofibroblasts are also αSMA‐positive. Since it has also been suggested that vascular smooth muscle cells could transdifferentiate towards a fibroblast phenotype through PDGFB signaling 56, we cannot exclude the possibility that some of the αSMA‐positive cells represent myofibroblasts. However, we can also not exclude that these myofibroblasts transdifferentiate back towards the pericyte phenotype to participate in vascular support. Therefore, we preferred αSMA staining over, for example, NG2 staining. The facts that we did observe αSMA^+^ cells lining VM^+^ structures and that NG2 positivity could be detected at sites distant from CD31^+^ vessels do suggest that αSMA^+^ cells that are not associated with normal blood vessels are likely to represent pericytes.

Collectively, our data demonstrate that aggressive VM^+^ tumor cells can mirror the behavior of regular blood vessels by producing PDGF and recruiting pericytes to support the formation of vascular‐like networks. Similarly as described for normal vessels, the perivascular cells stabilize the tumor cell‐lined vessels and stimulate the deposition of matrix proteins for structural support. Our data further show that the recruitment of perivascular cells is mediated through PDGFB signaling, which can be blocked by targeting PDGF receptors. These findings extend the observations that interfering with PDGF signaling could provide therapeutic benefit for different cancer patients 36; for example, for the treatment of patients with aggressive tumors that are refractory to angiostatic therapy. Moreover, a drug is available that has already passed phase I safety trials, i.e. imatinib. With the increasing interest in repurposing old drugs, this provides opportunities for the treatment of patients with aggressive VM^+^ tumors. In this context, it is noteworthy to emphasize that our current findings further contribute to the concept that pericytes are targets for cancer therapy as they play a key role in multiple vascular processes that are linked to tumor aggressiveness, including tumor angiogenesis, vascular mimicry, and vascular co‐option. For example, Cheng *et al* reported that glioma stem cells were able to generate pericytes to support tumor vascularization and tumor progression 57. In addition, Caspani *et al* showed that vascular co‐option in glioblastoma is dependent on pericytes 58. Since vascular co‐option and the related process of pericyte mimicry are also linked to tumor metastasis 59, 60 and resistance to angiostatic therapy 61, 62, it is tempting to speculate that malignant tissues rely on pericytes during mechanisms that contribute to tumor vascularization, metastasis, and even therapy resistance. Unraveling the pathways that underlie these pericyte‐mediated mechanisms will help to develop novel therapeutic opportunities.

## Author contributions statement

VLT, YWP, and AWG conceived and verified the study, were responsible for methodology, and carried out formal analysis. YWP and KLD conducted the investigation. KH, PP, JJvdO, VCT‐H, MJH, C‐HH, YC, PN‐S, AMC, and MR provided resources. AWG, YWP, and VLT wrote the original draft of the manuscript. All the authors reviewed, edited, and approved the final paper. VLT and YWP were responsible for visualization. AWG supervised the study.

**SUPPLEMENTARY MATERIAL ONLINE**

**Supplementary materials and methods**

**Figure S1.** Pericyte staining in human melanoma tissues

**Figure S2.** Quantification of intratumoral extravascular erythrocytes in melanoma tissues

**Figure S3.** Quantification of perivascular coverage (αSMA^+^ or αSMA^−^) of blood vessels (CD31^+^) in tumors from VM^+^ (C8161) or VM^−^ (OCM‐1) cell lines

**Figure S4.** PAS loop formation in cultured tumor cells in the presence or absence of HSVCs

**Figure S5.** Vascular‐like network formation in cultured tumor cells in the presence or absence of HVSCs

**Figure S6.** Effect of hypoxia and matrix on PDGFB expression in VM^+^ and VM^−^ cells

**Figure S7.** The effect of conditioned medium (CM) from VM^−^ C81‐61 or VM^+^ C8161 cutaneous melanoma cells on cellular organization of actin stress fibers in HVSCs

**Figure S8.** Vascular‐like network formation by B16F10 melanoma cells and the effect of PDGFB overexpression on the tumor vasculature in murine B16F10 tumors

**Figure S9.** Effect of interfering with PDGFB signaling on tumor vascularization in xenograft melanoma tumors

## Supporting information


**Supplementary materials and methods**

**Figure S1.** Pericyte staining in human melanoma tissues
**Figure S2.** Quantification of intratumoral extravascular erythrocytes in melanoma tissues
**Figure S3.** Quantification of perivascular coverage (αSMA^+^ or αSMA^−^) of blood vessels (CD31^+^) in tumors from VM^+^ (C8161) or VM^−^ (OCM‐1) cell lines
**Figure S4.** PAS loop formation in cultured tumor cells in the presence or absence of HSVCs
**Figure S5.** Vascular‐like network formation in cultured tumor cells in the presence or absence of HVSCs
**Figure S6.** Effect of hypoxia and matrix on PDGFB expression in VM^+^ and VM^−^ cells
**Figure S7.** The effect of conditioned medium (CM) from VM^−^ C81‐61 or VM^+^ C8161 cutaneous melanoma cells on cellular organization of actin stress fibers in HVSCs
**Figure S8.** Vascular‐like network formation by B16F10 melanoma cells and the effect of PDGFB overexpression on the tumor vasculature in murine B16F10 tumors
**Figure S9.** Effect of interfering with PDGFB signaling on tumor vascularization in xenograft melanoma tumors
