# Supplementary material for: Targeting PDGF‐mediated recruitment of pericytes blocks vascular mimicry and tumor growth
Source: J Pathol. 2018 Oct 30;246(4):447–58. doi: 10.1002/path.5152 (PMC6587443; doi:10.1002/path.5152)
Supplement: Supplementary file 1 — Supplementary materials and methods [file PATH-246-447-s001.docx]

**Targeting PDGF-mediated recruitment of pericytes blocks vascular mimicry and tumor growth**

**PDFG signaling promotes vascular mimicry**

**Thijssen VLJL *et al.* J Pathol 2018 (DOI: 10.1002/path.5152)**

**Supplementary materials and methods**

Reference numbers refer to the main text reference list

*PAS staining and IEE assessment*

For PAS staining, tissues were deparaffinized and rehydrated, and subsequently incubated with 0.5% periodic acid for 5 min. After washing with demineralized water, Schiff’s reagent was applied to the tissues for 10 min. PAS-stained tissues were counterstained with hematoxylin. PAS staining was scored manually by two independent observers using conventional light microscopy (Leica DM 4000B microscope equipped with a Leica DC 500 digital camera (Leica Microsystems, Amsterdam, The Netherlands). The presence of intratumoral extravascular erythrocytes (IEEs) was assessed on hematoxylin/eosin-stained tissues. For both PAS and IEEs, a dichotomous score was used, i.e. absent or present.

*Immunohistochemistry*

Formalin-fixed, paraffin-embedded melanoma tissues were cut into 5-μm-thick sections. Tissues sections were deparaffinized and rehydrated. Immersing the slides in 0.3% H_2_O_2_ in methanol for 20 min to block endogenous peroxidase activity. Heat-induced epitope retrieval was performed by autoclave treatment. After washing with PBS, non-specific antibody binding was blocked by PBS containing 5% bovine serum albumin (BSA). Perivascular cells were labeled with mouse anti-αSMA primary antibody (1:200, M0851; Agilent, Amstelveen, The Netherlands) for 1 h in PBS containing 0.5% BSA. For PDGFB detection, sections were incubated with rabbit anti PDGFB (1: 200, RB-9257-P0; Fisher Scientific, Landsmeer, The Netherlands) for 1 h in PBS containing 0.5% BSA. Visualization of primary antibody binding was established using the EnVision^TM^ Detection System (Agilent). Afterwards, tissues were counterstained with hematoxylin and eosin. For double staining of endothelial cells and perivascular cells, anti-CD31 antibodies (1:200, sc-1506; Santa Cruz Biotechnology, Heidelberg, Germany) and anti-αSMA antibodies (1:200, M0851; Agilent) were used, respectively. Primary antibodies were detected using either biotinylated rabbit anti-goat antibodies (1:1000, E0466; Agilent) visualized by streptavidin ABComplex/HRP (1:1000, K0377; Agilent), or biotinylated rabbit anti-mouse antibodies (1:1000, E0413; Agilent) visualized by streptavidin ABComplex/AP according to the supplier’s instructions (K0391; Agilent). In negative control stained sections, the primary antibody was omitted. The MVD was calculated as the average number of CD31^+^ vessels in 1–5 high-power fields (number of fields depended on the tumor/tissue size). Staining was scored by at least two independent observers. In addition to CD31+ alone, also the number of CD31^+^/αSMA^+^ vessels was scored in the same tissue area.

*Conditioned culture medium*

Conditioned culture medium was obtained as described for the ELISA experiments. To avoid inducing effects due to differences in pH the conditioned medium was concentrated twenty-fold by centrifugation though 10K Amicon Ultra Centrifugal Filter Units (Millipore/Merck, Amsterdam, The Netherlands). Conditioned medium was stored at -20°C and was not thawed more than once. In subsequent experiments, the conditioned medium was applied in a ten-fold dilution in serum-free medium.

*Reverse transcription–quantitative PCR (RT-qPCR)*

RT-qPCR was performed as described previously [63]. In brief, total RNA was isolated from the cells using RNeasy Mini Kit (Qiagen, Venlo, The Netherlands) including DNase I treatment. RNA quantity and quality were examined by spectrophotometry (Nanodrop ND-1000, Isogen Life Science, De Meern, The Netherlands). One microgram of total RNA was reverse-transcribed to cDNA using the iScript cDNA synthesis kit (BioRad, Veenendaal, The Netherlands). Real-time quantitative PCR mixes were composed of 1xSYBR Green (Bio-Rad), 400 nm of each primer and cDNA equivalent to 30 ng total RNA per reaction. Reactions were run in a C1000^TM^ Thermal System equipped with a CFX96^TM^ Real-Time System (BioRad). Data were analyzed using IQ5 software (BioRad). Melting curves were generated to monitor the production of single products. Geometric averages of C(t) values of the reference genes cyclophilin A (*PPIA*) and β-actin (*ACTB*) were subtracted from the C(t) values of the examined genes to generate dC(t) values. Relative expression was calculated as 2^-ΔC(t)^. No template controls, i.e. no addition of cDNA, served as negative controls.

*Fluorescent labeling of perivascular cells*

Perivascular cells were harvested and labeled with 5-(and 6-)-carboxyfluorescein diacetate, succinimidyl ester (CFSE) (Invitrogen/Fisher Scientific). CFSE was diluted in PBS to a concentration of 5 μm. Perivascular cells were incubated with CFSE for 10 min in a 37°C water bath after which complete medium was added in a 1:1 ratio. Following three washes in complete medium, cells were subsequently used in the Matrigel assay and sprouting assay.

*Sprouting assay*

Cells were suspended in RPMI-1640 containing 10% FBS and 20% methylcellulose (Sigma-Aldrich, M0512). Spheroids were formed by the hanging drop method in which each drop was formed by a volume of 25 µl containing 1000 cells. Combination spheroids were composed of 750 VM^+^ tumor cells added with 250 VM^-^ tumor cells or 250 perivascular cells. After overnight incubation at 37°C, spheroids were harvested and suspended in a collagen matrix containing 66.7% PureCol^TM^ collagen (Sigma Aldrich/Merck, Amsterdam, The Netherlands), 8.3% M199 10x cell culture medium (Gibco), 14.9% methylcellulose, 10% new born calf serum, and 0.1% heparin. The collagen gel was neutralized by the addition of 0.2 m NaOH. Following an incubation of 60 min at 37 °C, serum-free medium was added to the wells. Sprouting was imaged after 8 and 24 h using a Leica DMI3000B microscope. The images were quantified for the average sprout length using ImageJ (version 1.14o, https://imagej.nih.gov/ij/).
