# Supplementary material for: Targeting PDGF‐mediated recruitment of pericytes blocks vascular mimicry and tumor growth
Source: J Pathol. 2018 Oct 30;246(4):447–58. doi: 10.1002/path.5152 (PMC6587443; doi:10.1002/path.5152)
Supplement: Supplementary file 2 — Figure S1. Pericyte staining in human melanoma tissues Figure S2. Quantification of intratumoral extravascular erythrocytes in melanoma tissues Figure S3. Quantification of perivascular coverage (αSMA+ or αSMA−) of blood vessels (CD31+) in tumors from VM+ (C8161) or VM− (OCM‐1) cell lines Figure S4. PAS loop formation in cultured tumor cells in the presence or absence of HSVCs Figure S5. Vascular‐like network formation in cultured tumor cells in the presence or absence of HVSCs Figure S6. Effect of hypoxia and matrix on PDGFB expression in VM+ and VM− cells Figure S7. The effect of conditioned medium (CM) from VM− C81‐61 or VM+ C8161 cutaneous melanoma cells on cellular organization of actin stress fibers in HVSCs Figure S8. Vascular‐like network formation by B16F10 melanoma cells and the effect of PDGFB overexpression on the tumor vasculature in murine B16F10 tumors Figure S9. Effect of interfering with PDGFB signaling on tumor vascularization in xenograft melanoma tumors [file PATH-246-447-s002.pdf]

**Supplementary figures**

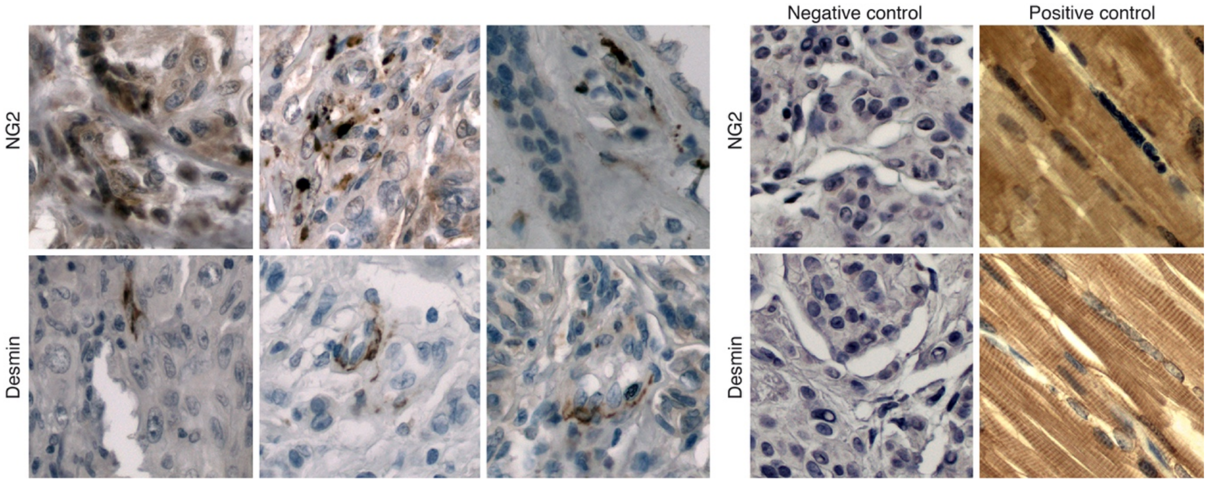


**Figure S1. Pericyte staining in human melanoma tissues.**

Representative images of immunohistochemical staining for NG2 (upper panels, brown) and desmin (lower panels, brown) in VM+ human melanoma tumor tissues. The panels on the right show negative controls (no primary antibodies used) and positive controls (muscle tissue).

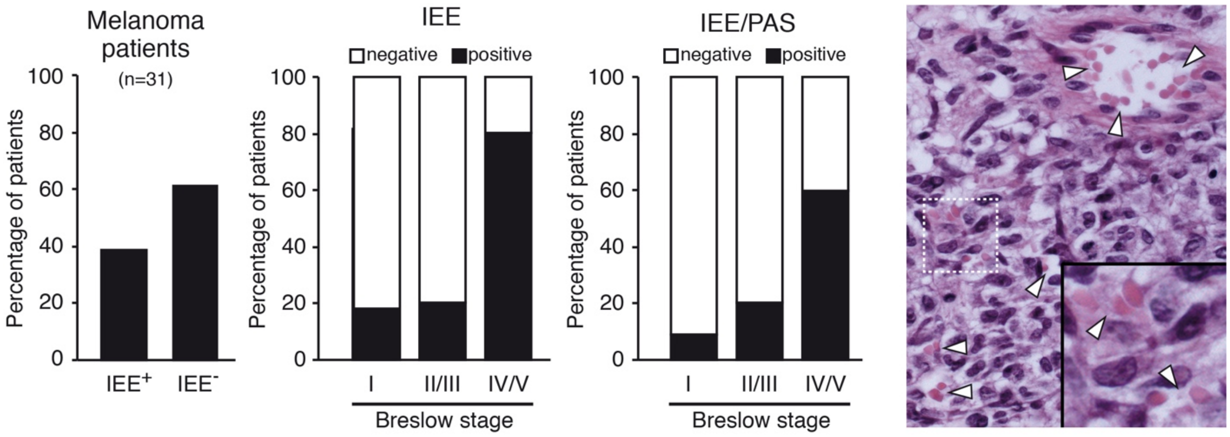


**Figure S2. Quantification of intratumoral extravascular erythrocytes in melanoma tissues.** Left bar chart shows the percentage of melanoma patients that present with intratumoral extravascular erythrocytes in their tumor tissue. The center left panel shows the percentage of IEE- and IEE+ patients classified according to Breslow stage. The center right panel shows the percentage of melanoma patients classified according to invasive activity (Breslow stage) which are either double positive or double negative for IEE and PAS. The image on the right shows an IEE+ region. Arrow heads point towards erythrocytes that are not associated with blood vessels but are located between tumor cells.

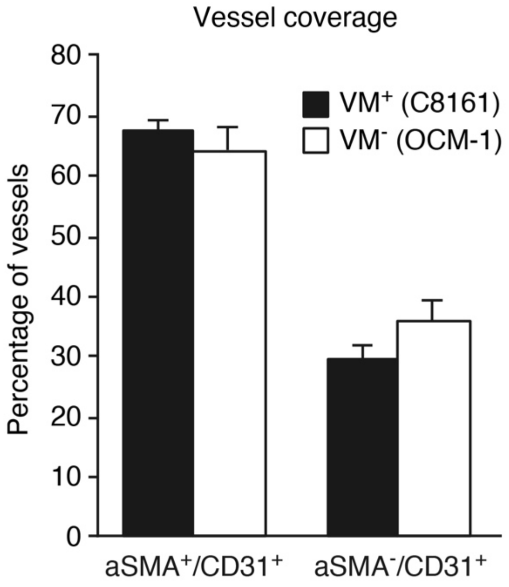


**Figure S3.** Quantification of perivascular coverage (αSMA+ or αSMA-) of blood vessels (CD31+) in tumors from VM+ (C8161) or VM- (OCM-1) cell lines.

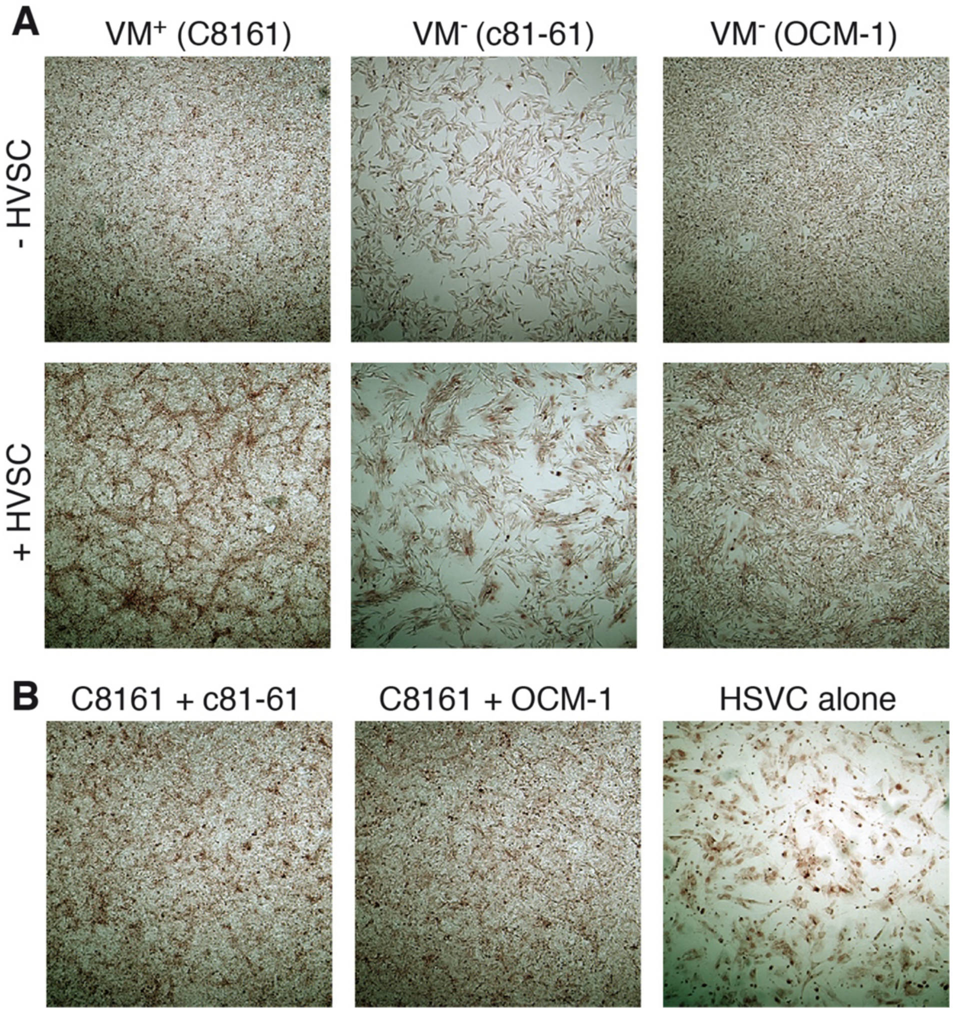


**Figure S4. PAS loop formation in cultured tumor cells in the presence or absence of HVSCs.**

(A) PAS stained co-cultures of both VM+ (left panels) and VM- (middle and right panels) cell lines in the absence (upper panels) or presence (middle panels) of HVSC perivascular cells. (B) PAS stained co-cultures of VM+ cells combined with VM- cells or HVSCs alone.

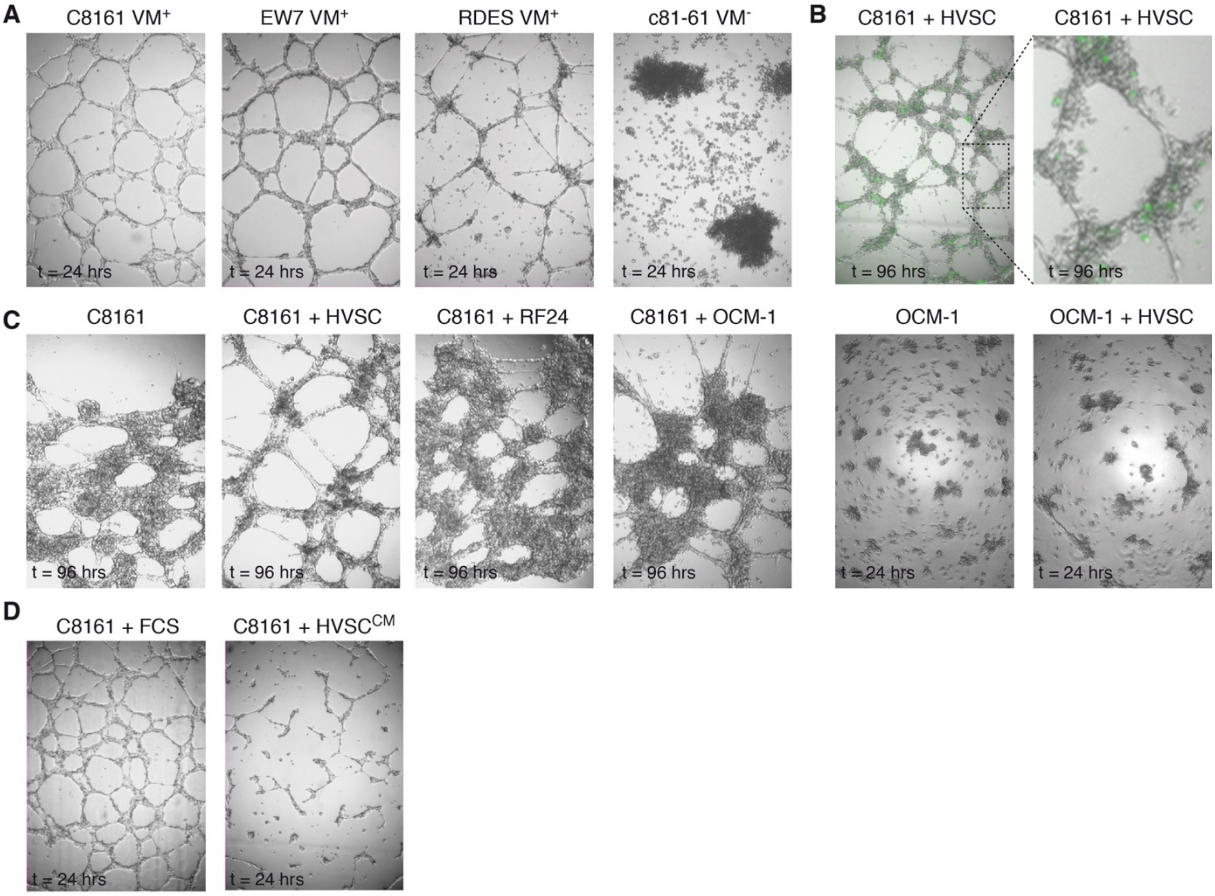


**Figure S5. Vascular-like network formation in cultured tumor cells in the presence or absence of HVSCs.**

(A) Formation of vascular-like networks on Matrigel by VM+ and VM- cell lines. (B) Co-localization of CFSE-labeled HVSCs (green) with vascular-like networks formed by C8161 cells at day 4. (C) Effect of different co-cultures on the stability of vascular-like networks formed on Matrigel by VM+ cell line C8161. Bottom two panels show absence of network formation by VM- cells even in the presence of HVSC. (D) Effect of normal culture medium or FCS-free conditioned medium of HVSCs on vascular-like network formation by C8161 cells.

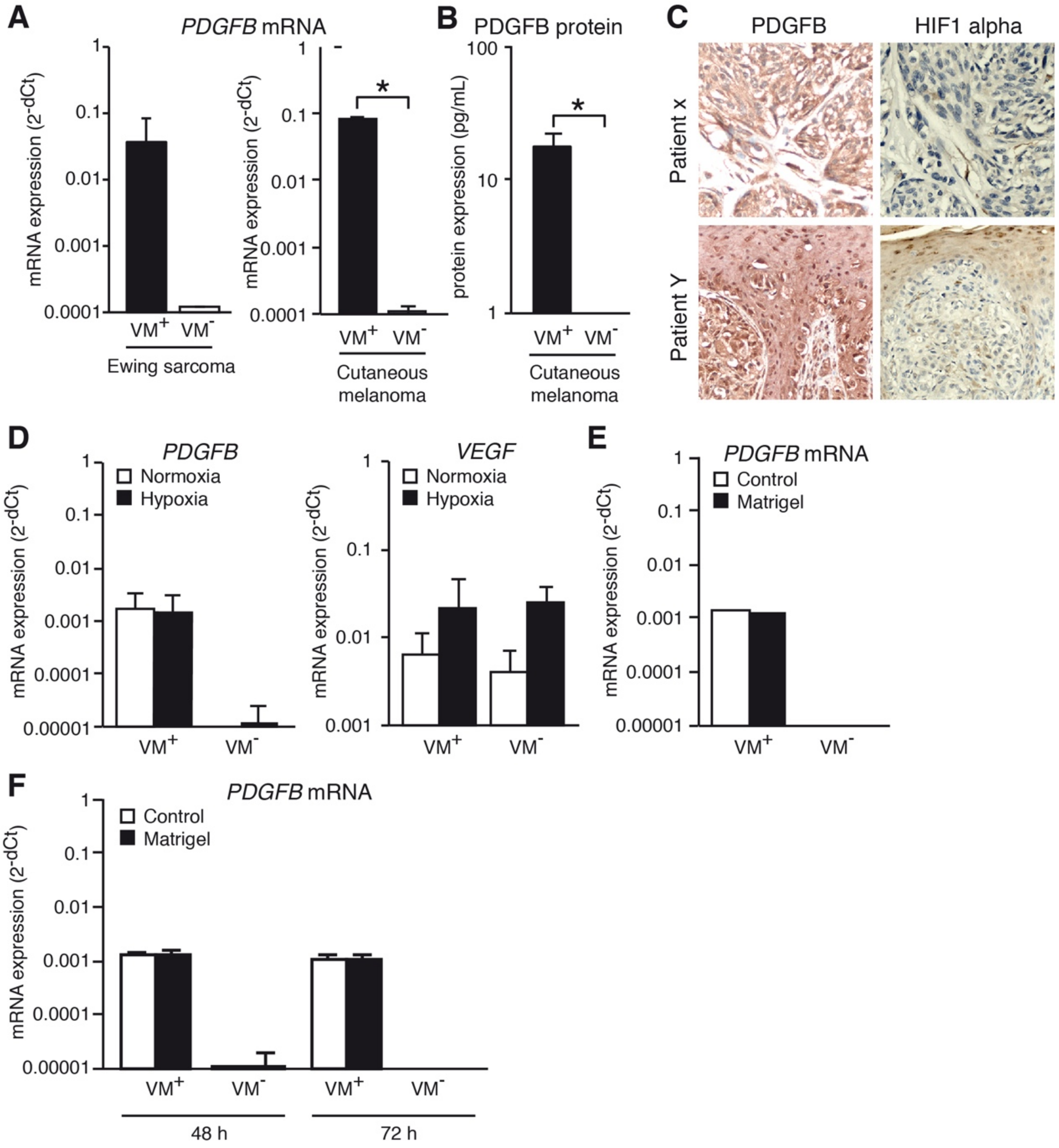


**Figure S6. Effect of hypoxia and matrix on PDGFB expression in VM+ and VM- cells.**

(A) The levels of *PDGFB* mRNA in different VM+ and VM- cell lines (C81-61, C8161). (B) The level of secreted PDGFB protein as assessed by ELISA in culture medium of VM+ and VM- cell lines (C81-61, C8161). (C) Representative images of PDGFB immunostaining staining (brown; left panels) and HIF1alpha immunostaining (brown; right panels) in consecutive sections of tumor tissue from patients with cutaneous melanoma. No apparent colocalization of PDGFB and HIF1alpha could be observed. (D) The levels of *PDGFB* mRNA (left bar chart) or *VEGFA* (right bar chart) in VM+ (C8161) and VM- (OCM-1) cells cultured under normoxic (white bars) or hypoxic (black bars) conditions for 3 days. (E) Similar to (D) for cells cultured on Matrigel for 24 h. (F) Similar to (D) for cells cultured on Matrigel for 48 or 72 h in the presence of HSVCs.

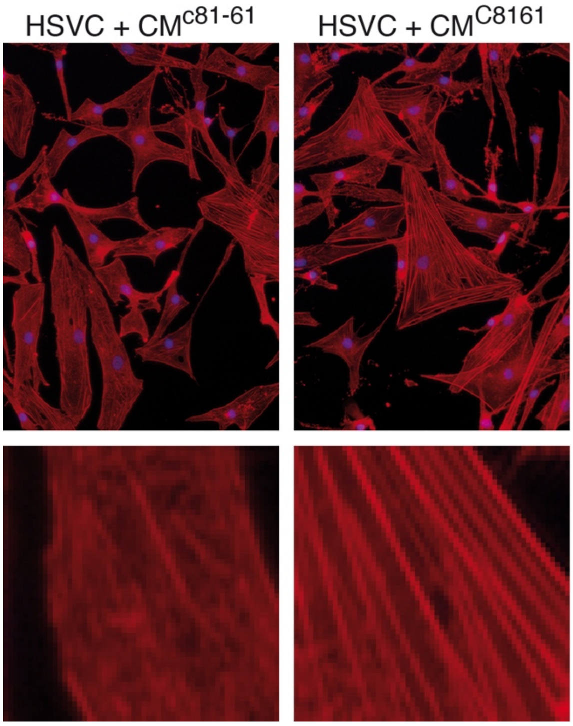


**Figure S7.** The effect of conditioned medium (CM) from VM- C81-61 or VM+ C8161 cutaneous melanoma cells on cellular organization of actin stress fibers in HVSCs.

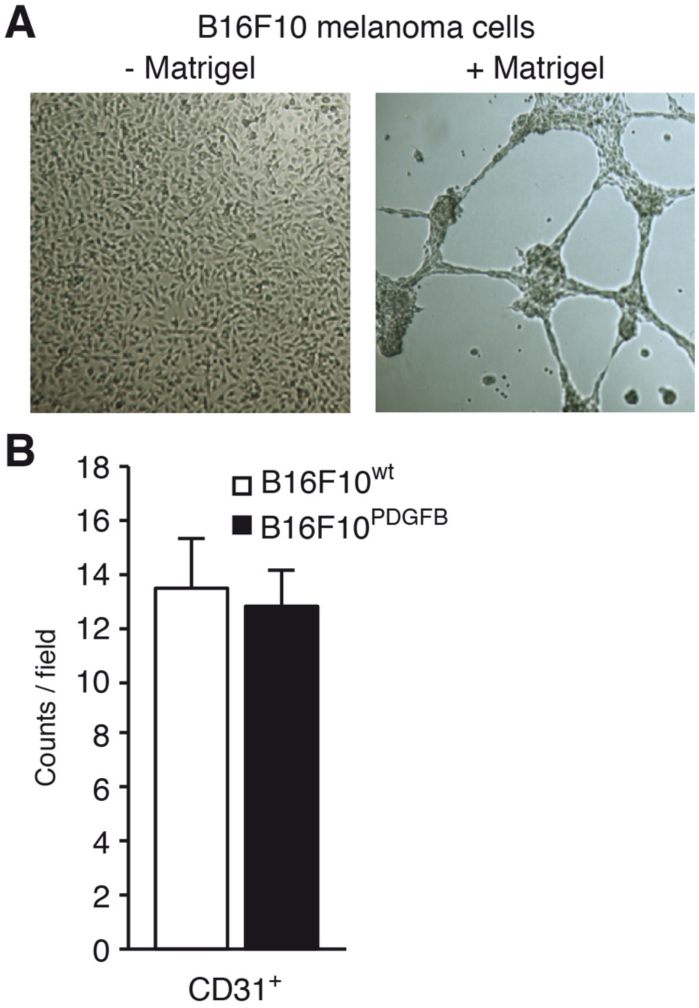


**Figure S8. Vascular-like network formation by B16F10 melanoma cells and the effect of PDGFB overexpression on the tumor vasculature in murine B16F10 tumors.**

(A) Formation of vascular-like networks by B16F10 melanoma cells on Matrigel (right panel). (B) Quantification of CD31+ endothelial cells in tumors from wildtype B16F10 cells or *PDGFB* overexpressing B16F10 cells.

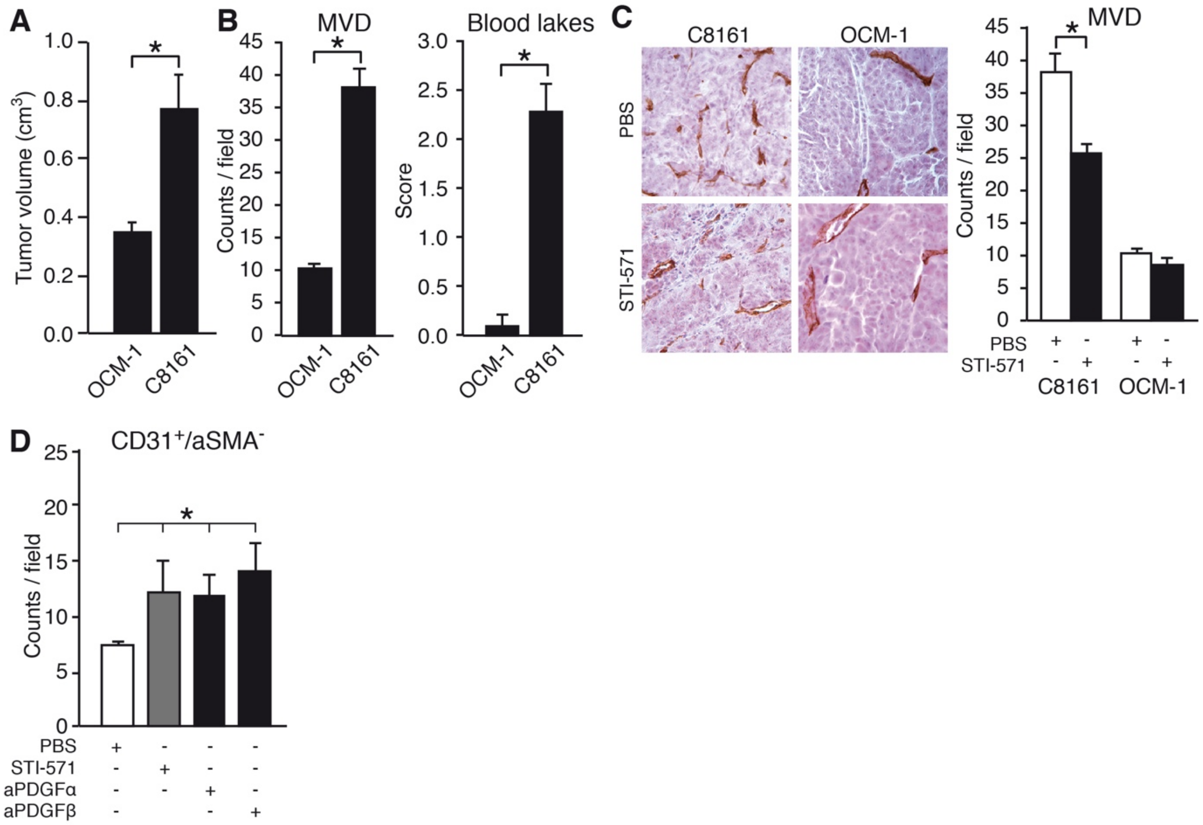


**Figure S9. Effect of interfering with PDGFB signaling on tumor vascularization in xenograft melanoma tumors.**

(A) Tumor volumes of VM+ (C8161) and VM- (OCM-1) xenograft tumors. * $p<0.05$. (B) Quantification of blood lakes and microvessel density in VM+ (C8161) and VM- (OCM-1) xenograft tumors. * $p<0.05$. (C) Representative pictures and quantification of CD31 staining in VM+ (C8161) and VM- (OCM-1) xenograft tumors treated with PBS or STI-571. * $p<0.05$. (D) quantification of CD31+ vessels not associated with αSMA+ perivascular cells in VM+ (C8161) xenograft tumors treated with PBS, STI-571 or blocking antibodies targeting either PDGF receptor alpha or PDGF receptor beta. * $p<0.05$ treatment versus PBS.
